# Room-temperature single-photon emitters in titanium dioxide optical defects

**DOI:** 10.3762/bjnano.9.100

**Published:** 2018-04-04

**Authors:** Kelvin Chung, Yu H Leung, Chap H To, Aleksandra B Djurišić, Snjezana Tomljenovic-Hanic

**Affiliations:** 1 School of Physics, The University of Melbourne, Parkville, VIC 3010, Australia; 2 Department of Physics, The University of Hong Kong, Pokfulam Road, Hong Kong; 3 Current address: The Nano and Advanced Materials Limited (NAMI), Science Park, Hong Kong

**Keywords:** fluorescence, optical defects, room temperature, single-photon emitters, titanium dioxide

## Abstract

Fluorescence properties of crystallographic point defects within different morphologies of titanium dioxide were investigated. For the first time, room-temperature single-photon emission in titanium dioxide optical defects was discovered in thin films and commercial nanoparticles. Three-level defects were identified because the *g^(2)^* correlation data featured prominent shoulders around the antibunching dip. Stable and blinking photodynamics were observed for the single-photon emitters. These results reveal a new room-temperature single-photon source within a wide bandgap semiconductor.

## Introduction

Single-photon sources offer non-classical states of light [1] and are a prerequisite for future quantum technologies [2]. There are many types of single-photon emitters that include molecules [3], trapped atoms [4], quantum dots [5] and defects in diamond [6]. More recently point defects of wide-bandgap semiconductors, such as zinc oxide (ZnO) [7–9] and silicon carbide [10], were shown to exhibit room-temperature single-photon emission. ZnO is the only metal oxide reported to host single-photon emitting defects at room temperature and was recently shown to exhibit stable fluorescence when uptaken into skin cells, making it a viable biomarker [11].

Titanium dioxide (TiO_2_) is a well-studied wide-bandgap semiconductor, its production cost is low and it is used as a white pigment in foods, cosmetics [12], textiles [13] and paints [14]. It has a relatively high refractive index of 2.3 at 550 nm [15] and recent work demonstrated its potential applications as novel optical material for waveguides and resonators [16–21]. TiO_2_ can be fabricated using many methods resulting in an abundance of nanostructures [22]. In nanoparticle form, TiO_2_ is a constituent of sunscreens [23–24]. Other applications also include elimination of environmental pollution [25–28], and energy [29] and sensing applications [30–32]. Semiconductor defects have been touted as an promising platform for the development of a quantum computer in the solid state [33] in which the usage of TiO_2_ could be possible with further research into its quantum and physical properties.

TiO_2_ crystallises into three main forms: anatase, rutile and brookite [34]. Defects can be introduced during fabrication or are intrinsic to the crystallographic structure. Extensive work on TiO_2_ surface defects [35] has come from the need to progress catalytic reactions. Point defects within the TiO_2_ include interstitials and vacancies [36–38].

The defects are responsible for visible photoluminescence (PL) in TiO_2_ and have been observed in thin films [39–41], nanocrystals/nanoparticles [42–47], nanorods [48], nanotubes [49–51], nanosheets [52], nanoribbons [53] and fibres [54]. In material sciences, the PL spectrum of a sample is obtained by large spot size excitations, e.g., Amekura et al. investigated the PL from ZnO nanoparticles with a spot size of approximately 4 mm [55–56]. This spot size constitutes an ensemble measurement where PL from many defects is sampled. Therefore, single defects and their emission peaks cannot be resolved. As a comparison, the spot size used to excite the defects in this work was 280 nm.

This paper presents exploratory optical studies of various TiO_2_ morphologies. For the first time, defects in TiO_2_ thin films and nanopowders exhibited single-photon emission. Standard characterisation measurements of fluorescence microscopy, correlation measurements, PL spectra and photodynamics are presented.

## Experimental

### Electron-beam deposition of TiO_2_ thin films

The films were fabricated via e-beam deposition in high vacuum with the substrate temperature set to 200 °C during deposition. Subsequently, the samples were left untreated (“non-annealed”), or were annealed in air in a tube furnace at two temperatures: 450 and 850 °C. The ramping rate of the furnace was 5 °C/min. These samples are labelled NA-TiO_2_, a-450 °C-TiO_2_, and 850 °C-TiO_2_. A fourth film sample was also fabricated in a similar manner except that the substrate temperature was set to 160 °C and annealed at 450 °C in the same manner as a-450 °C-TiO_2_. This sample is labelled b-450 °C-TiO_2_.

### Preparation of TiO_2_ nanopowder samples

Two nanopowder phases, anatase and rutile (MTI Corporation) were used. The anatase (rutile) has a purity of 99% with an average particle size of 30 nm (45 nm). Four nanopowder samples were prepared: anatase and rutile suspended in deionised (DI) water, and anatase and rutile suspended in isopropyl alcohol (IPA). For the nanopowder–DI water mixture, 21.0 (20.6) ± 0.2 mg of anatase (rutile) nanopowder was suspended in 10 mL of DI water. Similarly for the nanopowder–IPA mixture, 19.9 (20.1) ± 0.2 mg of anatase (rutile) was suspended into 10 mL of IPA. The mixtures were ultrasonicated for 5 min to disperse the nanopowder evenly into both DI water and IPA yielding concentrations of approximately 2 mg/mL.

Each mixture was dripped with a pipette onto a silicon wafer whilst on a hotplate (60–90 °C) to evaporate the solvent, leaving a layer of nanopowder. This was repeated until an obvious white layer on top of the wafer was deposited. Smaller amounts of nanopowder can be used, but as it will be seen in the section “Results and Discussion”, it is preferable to obtain a clear indication of deposited nanopowder on the substrate.

### Confocal microscopy

**Figure 1:**
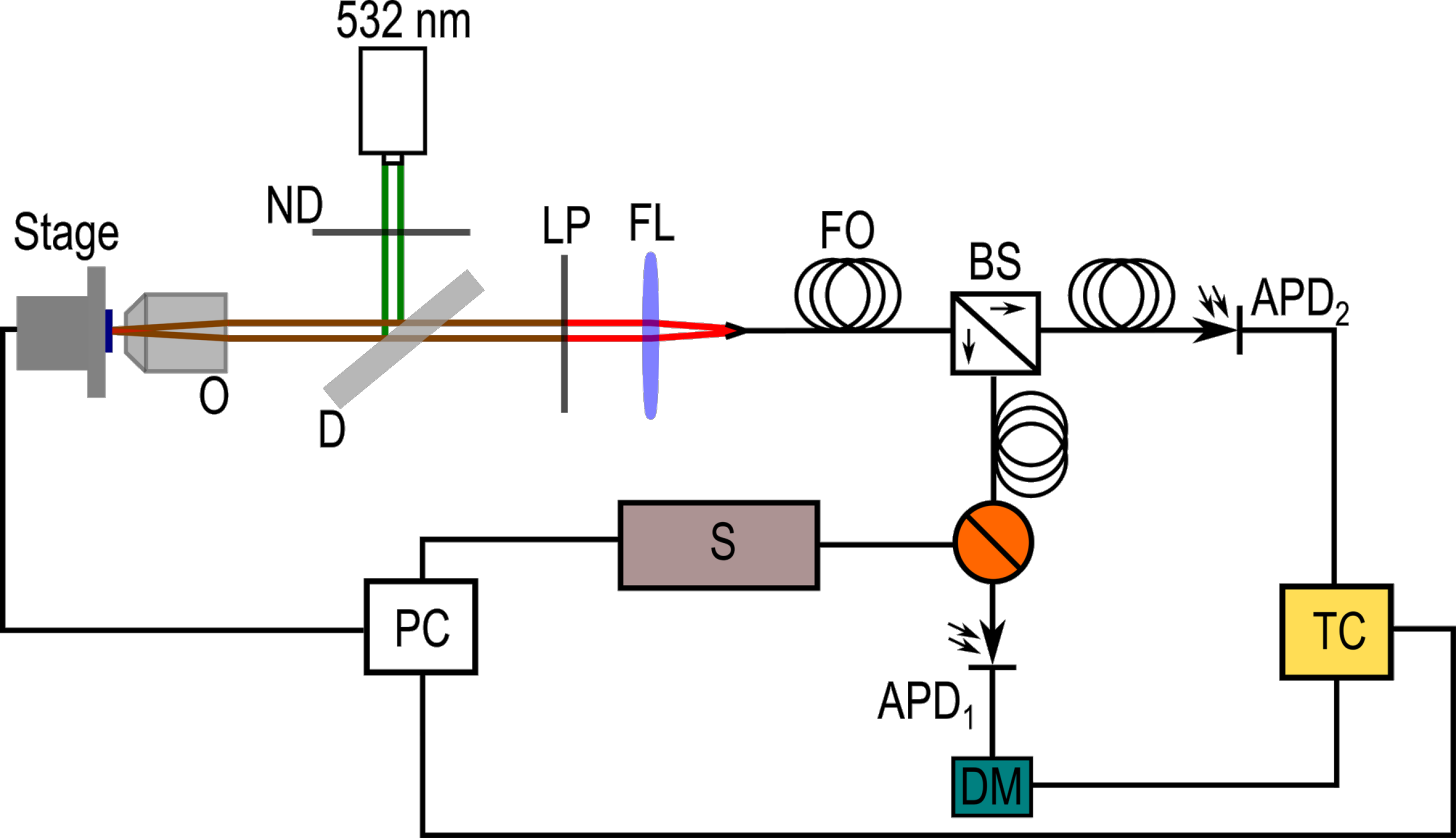
The experimental setup of the scanning confocal microscope used for investigating TiO_2_ defects.

Figure 1 is a schematic of the scanning confocal microscope used to investigate the TiO_2_ defects. The samples were illuminated by a frequency-doubled Nd:YAG laser (λ = 532 nm) the intensity of which was controlled with a neutral density filter (ND). The laser is reflected off a dichroic mirror (DM) and focussed onto the sample with a 100× (0.95 NA) air objective (O). The lateral resolution and diffraction-limited spot size was approximately 280 nm (in the plane of the substrate). The sample is mounted on a piezoelectric controlled stage with 100 μm travel. The fluorescent light is recollected through O and passes through a 560 nm long-pass filter (LP) to filter out the excitation laser. A converging lens (FL) focusses the fluorescent light into an optical fibre (FO), which acts as the confocal pinhole. The fluorescence signal is fibre-optically split 50:50 (BS) incident upon two avalanche photodiodes (APD_1,2_, Perkin Elmer SPCM-AQRH-14-FC: timing resolution = 350 ps at 825 nm). The system can be switched (orange junction) between two main configurations for taking spectra (S) and performing Hanbury Brown–Twiss (HBT) interferometry, which uses a delay module (DM) and a time-correlated single-photon counting system (TC). A computer (PC) was used to control the stage, spectrometer and correlation data acquisition parameters.

## Results and Discussion

### Confocal microscopy of various TiO_2_ morphologies

**Figure 2:**
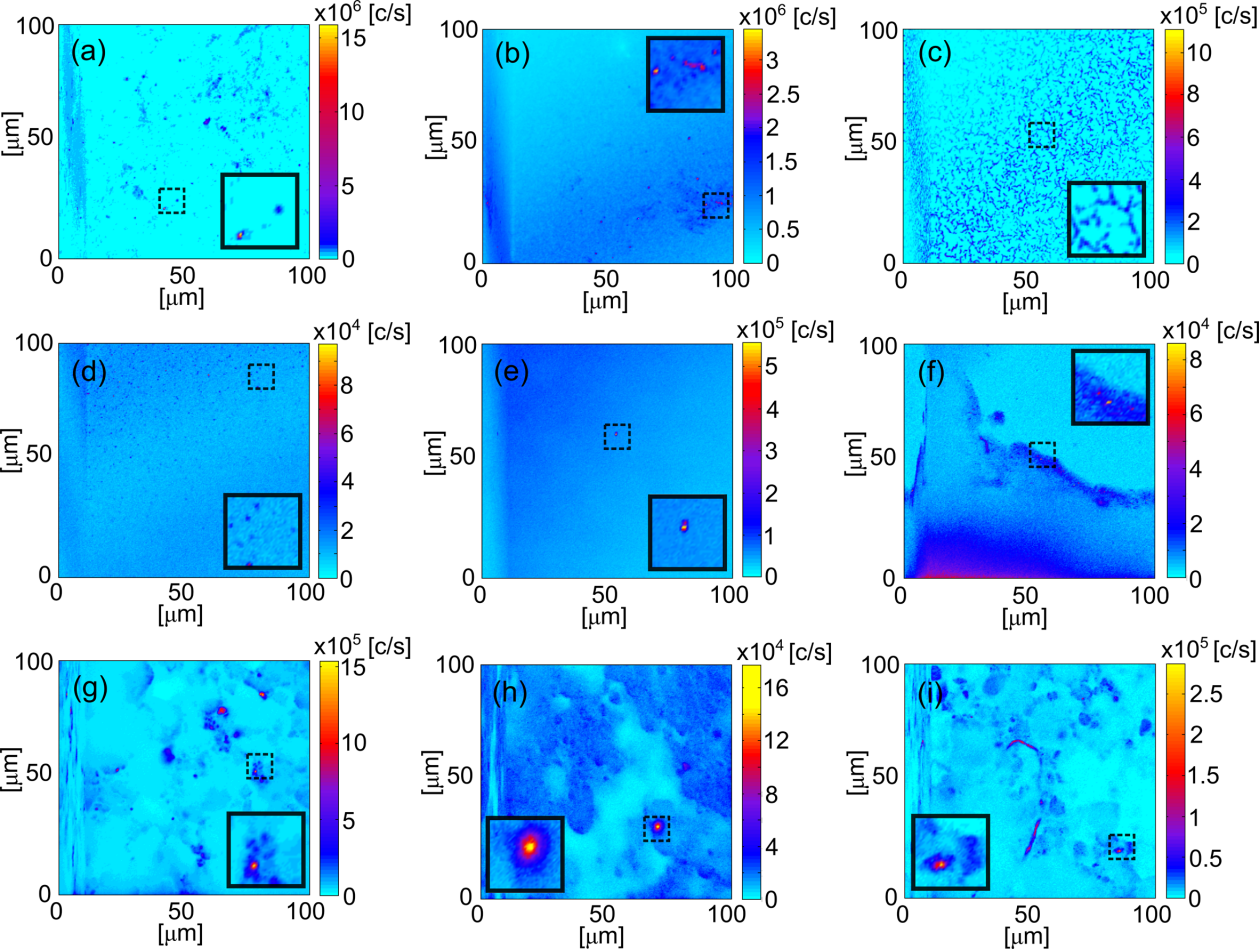
Representative 100 × 100 μm^2^ confocal scans of TiO_2_ morphologies. E-beam deposited thin films: (a) NA-TiO_2_, (b) a-450 °C-TiO_2_, (c) 850 °C-TiO_2_ and (d) b-450 °C-TiO_2_. (e) Single crystal rutile(001) with edges of 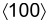. Nanopowder samples: (f) rutile + DI water; (g) anatase + DI water; (h) rutile + IPA and (i) anatase + IPA. The colour bars represent the count rate at the detector for each sample. The solid line black box represents the magnified region of the dashed line black box with 10 × 10 μm^2^ area.

The various TiO_2_ samples were investigated at room temperature using scanning confocal microscopy. This form of microscopy allows for high-resolution images that resolve fluorescence signals from individual defects. The motivation in exploring different TiO_2_ morphologies was to determine if room-temperature single-photon emitters exist. Figure 2 shows representative 100 × 100 μm^2^ confocal scans of the TiO_2_ samples including thin films, single crystal and nanopowders. The films were synthesized as described above, single crystals and nanopowders were purchased (MTI Corporation).

Thin films annealed at various temperatures, NA-TiO_2_, a-450 °C-TiO_2_, b-450 °C-TiO_2_ and 850 °C-TiO_2_, were investigated. The expected TiO_2_ phases were an amorphous phase in the untreated sample, anatase in a-450 °C-TiO_2_ and rutile in 850 °C-TiO_2_ [57]. The confocal scans in Figure 2a–d show fluorescent features in all samples. The untreated and 450 °C films show point-like fluorescent features, whereas the 850 °C reveals filament-like fluorescent structures. The total area scanned for each thin film sample was approximately 1 × 1 cm^2^.

The single-crystal rutile was 10 × 10 × 1.0 mm^3^ in size. It had an orientation of (001) with edges of 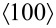, and a purity greater than 99.99%. Figure 2e is a representative confocal scan and is devoid of fluorescent features.

Anatase and rutiles nanopowders were dispersed into water and IPA. Figure 2f–i show fluorescent features from point-like objects alongside large areas of contrast, due to defects within the nanopowders and residue of the solvent, respectively. The total area scanned for each nanopowder sample was approximately 0.5 × 0.5 cm^2^.

Each sample was investigated for fluorescing defects, which were further examined for single-photon emission by observing its photon statistics. Once a single-photon emitter was identified, the defect was characterised by obtaining its PL spectrum and recording the photodynamics of its count trace. Fluorescence was observed in all the morphologies. However, single-photon emission was only observed in two morphologies, the characterisation of these single-photon emitting defects is presented in the next two subsections.

The single-crystal TiO_2_ morphology was produced via the floating-zone growth process. The production of TiO_2_ nanoparticles in industry involves wet chemical processes. For both morphologies, thermal treatment is also required. To the best of our knowledge, we can only conclude that we did not observe single-photon emission in some morphologies probably due to different fabrication methods and annealing temperatures. It can be inferred that for these two morphologies there is a pure non-radiative decay mechanism. This topic is beyond the scope of the current work, which focusses on the optical regime and single-photon emission. Morfa et al. [7] observed a dependence of the creation of defects in ZnO nanoparticles on the annealing temperature.

### Single-photon emission in TiO_2_ thin films

**Figure 3:**
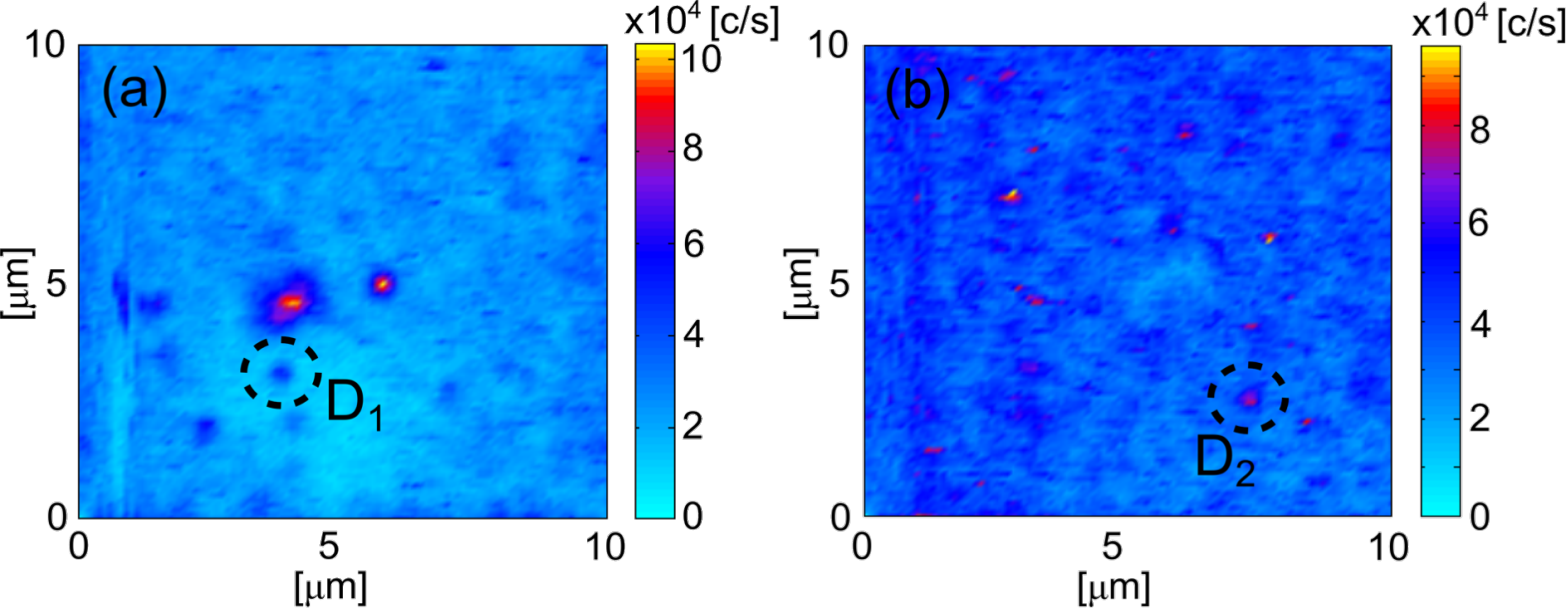
10 × 10 μm^2^ confocal scans of two single-photon emitters (a) defect D_1_ and (b) defect D_2_ found on the a-450 °C-TiO_2_ sample. The colour bars represent the count rate at the detector.

The a-450 °C-TiO_2_ sample exhibited single-photon emission. For a given arbitrary coarse scan of 100 × 100 μm^2^, fluorescence spots were observed to be sparse in character. Figure 3 shows characteristic 10 × 10 μm^2^ confocal scans of the sample. Two defects, D_1_ and D_2_, were identified and characterised. The defects were found during unique coarse scans, which were separated in distance by hundreds of micrometres.

**Figure 4:**
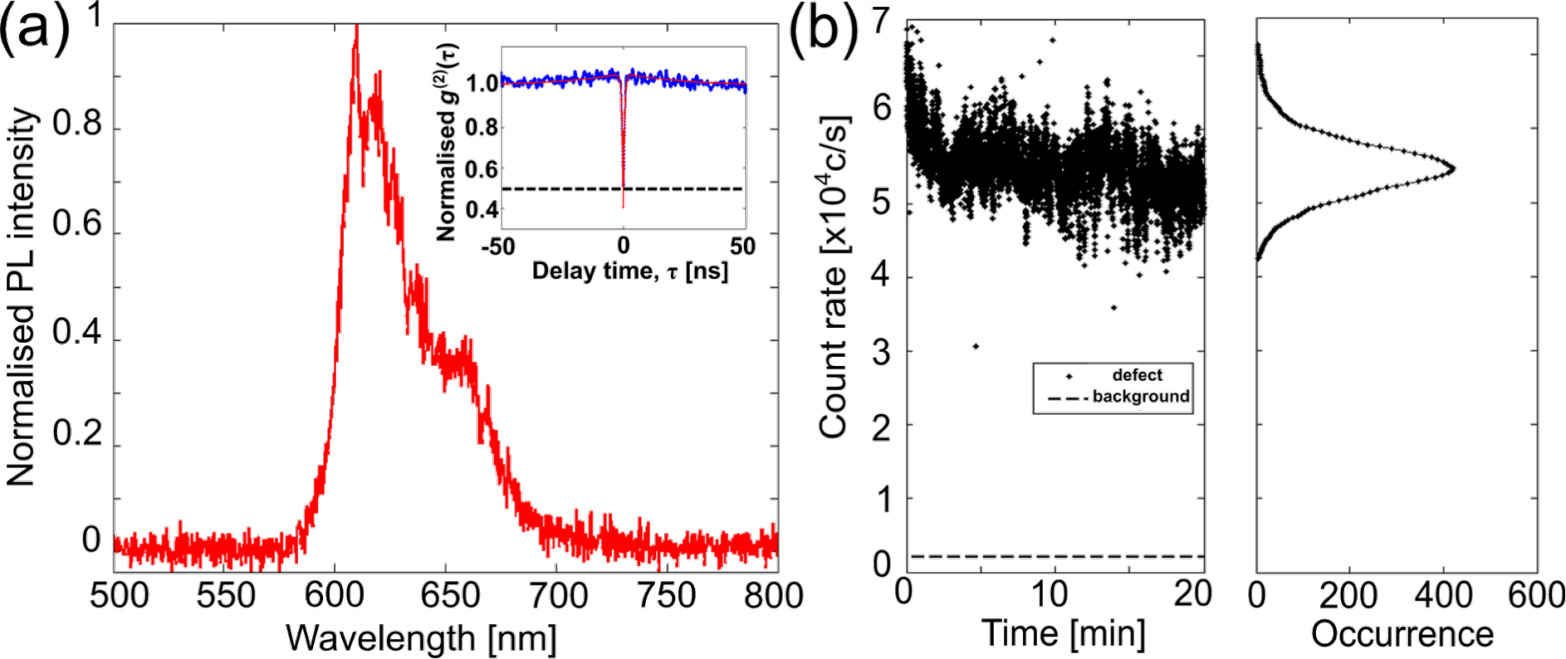
Characterisation results of defect D_1_, shown in Figure 3a, found in the a-450 °C-TiO_2_ sample. (a) Normalised PL intensity with an inset of the normalised *g^(2)^* data (blue points) with a three-level model fit (red line) at 82 ± 1 μW pump power. The time bin was 64 ps, with an integration time of 1200 s, the count rates at the detectors was 41.7 × 10^3^ and 40 × 10^3^ c/s with a background of 2 × 10^3^ c/s. The dashed line represents the normalised *g^(2)^*(τ) = 0.5. (b) The count trace and histogram for 82 μW pump power. This histogram had the count rate binned into 511 c/s. The background count rate is indicated by the dashed line.

D_1_ and D_2_ were not necessarily the brightest defects in the scans with much larger count rates observed for other fluorescent features. The other bright features could be other defects or contaminations. Defects D_1_ and D_2_ had their fluorescence monitored via a HBT interferometer to quantify their photon statistics. For a single-photon emitter, the second-order correlation function needs to satisfy the inequality: *g^(2)^*(τ = 0) *<* 0.5, where τ is the delay time electronically imposed to one of the detectors in the HBT setup. For D_1_, a three-level model was chosen to fit normalised *g^(2)^* data. It has the form [6,58]: *g^(2)^*(τ) = *A* − *B*·exp(−κ_21_)τ + *C*·exp(−κ_23/31_)τ, where *A*, *B* and *C* are fitting coefficients. The excited state and non-radiative decay rates are represented by κ_21_ and κ_23/31_*,* respectively. An appropriate fit to all parameters was achieved by minimising the least squares error between the three-level model and the normalised *g^(2)^* data. This fit is shown in the inset of Figure 4a. At τ = 0, the second-order correlation function was 0.40 ± 0.05, which satisfies the inequality for a single-photon emitter, i.e., the emission events were antibunched. At a pump power of 82 ± 1 μW, the excited and non-radiative lifetimes were calculated to be 0.52 ± 0.01 ns and 25.88 ± 5.25 ns, respectively. The normalised *g^(2)^* data was smoothed (moving average filter) before the lifetimes were calculated. The coefficients of the three-level model (*A*, *B*, *C*, κ_21_ and κ_23/31_) were determined by minimising the least squared error between the model and normalised data. These lifetimes are comparable to single-photon emission of ZnO defects [7–9].

Upon confirmation of single-photon emission, the PL spectrum of D_1_ was acquired and can be seen in Figure 4a. The PL spectrum of the defect shows red fluorescence between 600 and 700 nm. There were three resolvable peaks at 610, 619 and 630 nm. Red fluorescence has been attributed to under-coordinated Ti^3+^ ions in atomic layer deposited films [41], electrons trapped at surface defect sites [59] and surface oxygen vacancies on anatase nanocrystal films [60]. Similar photoluminescence in the red was also observed from ZnO defects [7–9].

The photodynamics of D_1_ was also recorded and can be seen for a pump power of 82 μW in Figure 4b. The time trace shows photostability with no obvious fluorescence intermittency, i.e., blinking. Subsequent measurements of D_1_ could not be conducted beyond 118 μW due to the defect photobleaching. This was confirmed by re-scanning the area of interest during which the original bright spot on the confocal image, indicative of a fluorescing defect, had ceased to the background count rate (3 × 10^3^ c/s). A histogram of the count rates at 118 μW shows a peak frequency above the background count rate. This behaviour can only be due to photoionisation of D_1_. The fluorescence photostability was different from previous works on ZnO defects in which fluorescence intermittency was observed [7–9].

**Figure 5:**
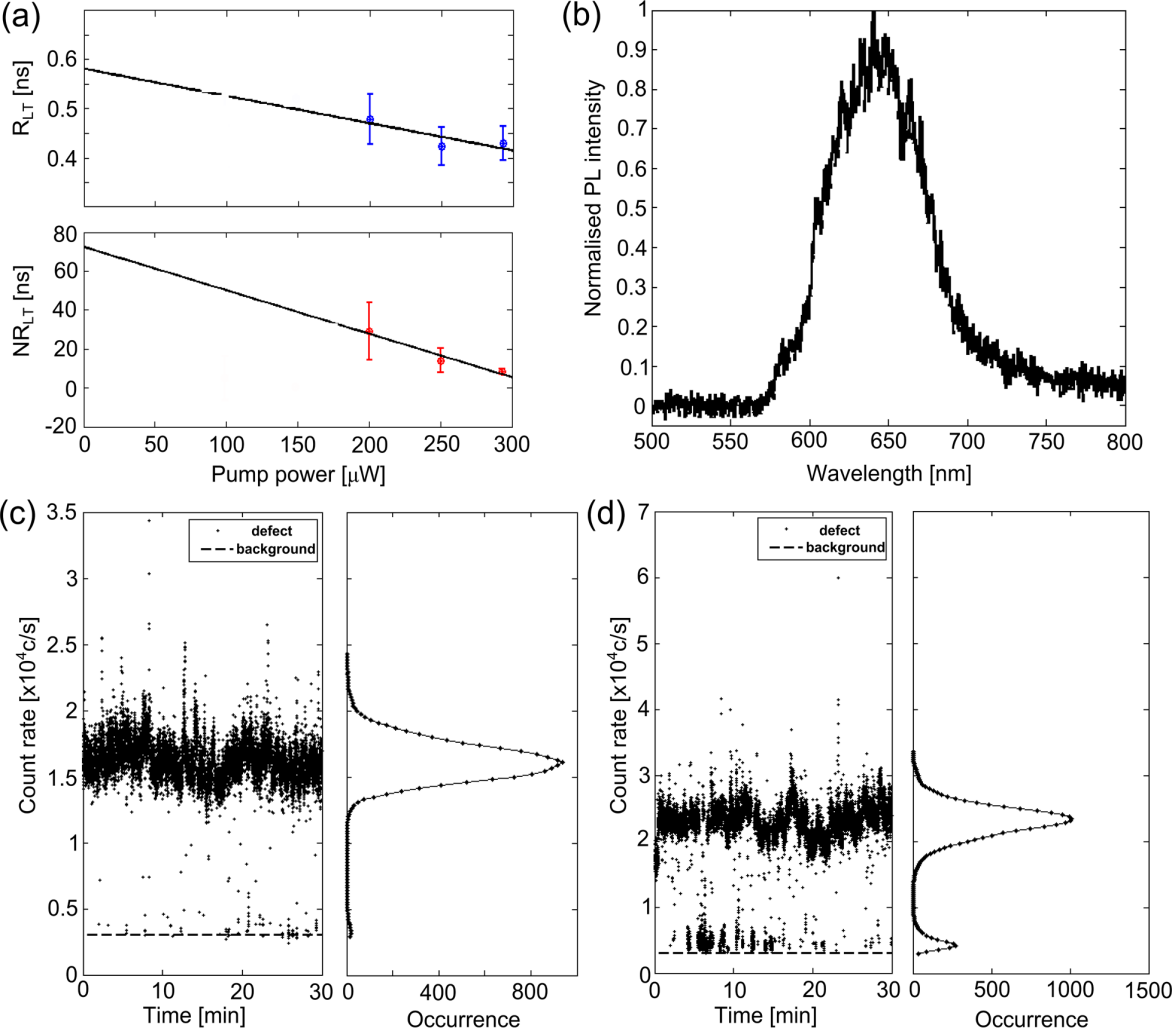
Characterisation results of defect D_2_. (a) The excited-state (R_LT_) and non-radiative (NR_LT_) lifetimes calculated using a three-level model (with 95% confidence intervals in the uncertainty bars). The black line represents a linear fit for the intrinsic lifetimes using pump powers of 200, 250 and 293 μW. (b) Normalised PL intensity. The time traces and histograms at pump powers: (c) 99 ± 0.5 μW (histogram binned into 308 c/s), (d) 148 ± 0.5 μW (histogram binned into 533 c/s). The dashed line represents the background count rate of 3 × 10^3^c/s.

The characterisation of D_1_ was only partially completed because the defect photobleached during data acquisition. Therefore, an intrinsic lifetime could not be determined. Only one D_1_-type defect was observed in this study. It was not anticipated that the defect would photobleach at such low pump powers. However, for defect D_2_ bleaching did not occur and the intrinsic lifetimes could be calculated. The correlation data of defect D_2_ was fitted with a three-level model and the inset of Figure 5b shows a fit at a pump power of 293 μW. The excited-state (R_LT_) and non-radiative (NR_LT_) lifetimes were calculated for various pump powers incident upon D_2_ and are shown in Figure 5a.

Linear fits of the lifetimes as a function of pump power allows for the calculation of the intrinsic lifetimes, which are represented by the zero-power intercept. The linear fits omit the pump powers of 99 and 148 μW due to their poor statistics, there was a large variance in the correlation data away from the centre of the antibunching dip. A moving average filter was applied to the data to obtain a smooth response to assist with the fitting, without success. Therefore, the two low pump powers were considered to be outliers. The intrinsic lifetimes were calculated using pump powers of 200, 250 and 293 μW. The intrinsic excited-state and non-radiative lifetimes were calculated to be 0.58 ns and 72.44 ns, respectively. These values are similar to values of ZnO defects [7]. Figure 5b is the spectrum of D_2_, which shows red fluorescence between 575 and 800 nm with a broad peak centred around 640 nm. Compared to the spectrum of D_1_ it has different spectral features, which means that D_1_ and D_2_ are two chemically different defects.

Figure 5c is the count trace and histogram observed for D_2_ at 99.0 ± 0.5 μW pump power exhibiting relative photostability. When the power is increased to 148.0 ± 0.5 μW, the defect exhibits blinking between two distinct levels of an “off” and an “on” state, i.e., a ground state and a bright state, which can be seen in Figure 5d. D_2_ showed robustness to permanent photoionisation, it did not photobleach after a long data acquisition period totalling 3 h. Furthermore, the photodynamics for D_2_ show a contrast to the behaviour of D_1_, which indicates that different defects were obtained in the films. It must be noted, that single-photon emitters in the a-450 °C-TiO_2_ sample were quite rare given the many fluorescing features on a 100 × 100 μm^2^ scan. Investigating many circular-like fluorescing features within a smaller region of 10 × 10 μm^2^, qualitatively, the D_1_ and D_2_ defects were the only features to exhibit single-photon emission. These two single-photon emitting defects represent 5% of the total number of fluorescing features investigated.

A previous work by Morfa et al. [7] on single-photon emitting defects in ZnO films showed that the annealing temperature plays an important role in the creation of defects. In our work, the films that were not annealed and those annealed at 850 °C exhibited no single-photon emission.

### Single-photon emission in TiO_2_ nanopowders

**Figure 6:**
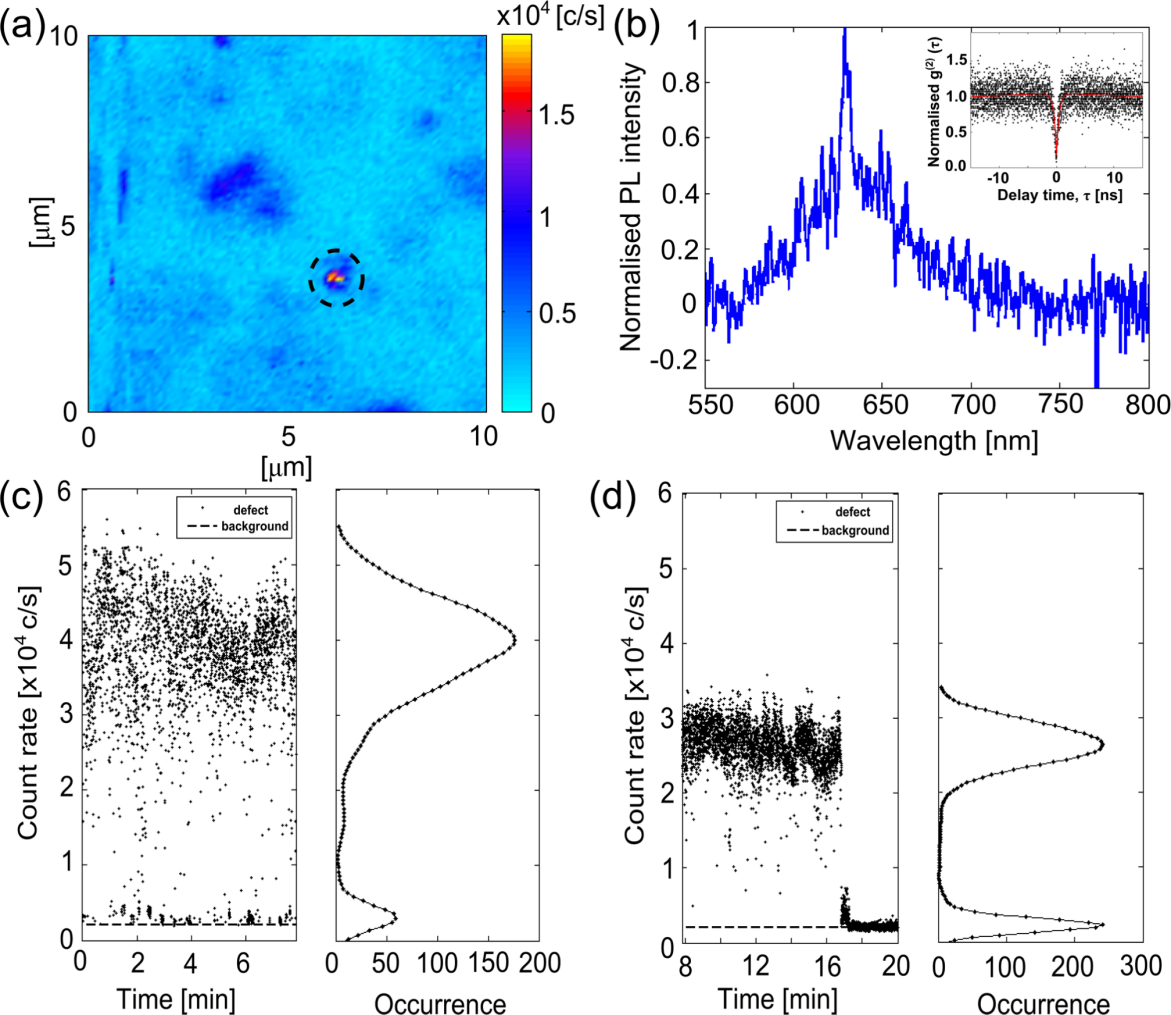
Characterisation results of a defect found in the sample of TiO_2_ anatase nanopowder and IPA. (a) 10 × 10 μm^2^ confocal scan indicating the defect with a dashed circle. The colour bar represents the count rate at the detector. (b) Normalised PL spectrum with an inset of a three-level model fit (red line) of the normalised *g^(2)^* data (black points). The fit parameters had count rates of 25.0 × 10^3^ c/s and 24.5 × 10^3^ c/s at each detector, a background count rate of 2 × 10^3^ c/s and integration time of 1200 s. (c, d) Count traces and histograms of the single-photon emitter for two periods of time (0–8 min and 8–20 min, respectively). The histograms in (c) and (d) were binned into 982 c/s and 505 c/s, respectively. The background count rate is indicated by the dashed line. The pump power for the results in panels b–d was 41.9 ± 0.1 μW.

Single-photon emission was also observed in the sample of anatase nanopowder and IPA (see Figure 2i for a representative confocal scan). The defect shown in Figure 6a was found to exhibit single-photon emission with *g^(2)^*(0) = 0.17 ± 0.04. The normalised *g^(2)^* data was fit with a three-level model (Figure 6b, inset) and the excited-state and non-radiative lifetimes were calculated to be 0.46 ns and 19.49 ns, respectively, similar to single-photon emitters in a-450 °C-TiO_2_ films. A three-level system was used because there were prominent shoulders around the antibunching dip in the normalised *g^(2)^* data. Interestingly, the confocal map of the single-photon emitting defect in the nanopowder shows the possibility of two entities. It can be seen there are two distinct spots, this is a unique feature to the nanopowder and is not evident in the other single emitters presented in this study.

Figure 6b is the spectrum of the defect, which shows red emission with a peak around 629 nm. The low signal-to-noise ratio was counteracted by increasing the exposure time at each step for the range of 550–1000 nm. The complete spectrum was combined from three separate exposures. The exposure time was set to 120 s. Longer times were not used but due to the chance of the defect permanently photobleaching at longer exposure times. The emission bandwidth matches the previous defects found on films. It is typical for the sub-bandgap excitation at 532 nm wavelength used here. Previous work by Mathew et al. [61] on TiO_2_ anatase colloidal nanoparticles has shown fluorescence spectra with distinct emission peaks in the visible spectrum between 400 and 600 nm. Mathew et al. also attributed their visible emission to surface states originating from oxygen vacancies associated with Ti^3+^ ions. The work of Zhang et al. [42] on anatase nanocrystals fabricated through a chemical process could not conclude definitively the origin of their broad visible emission band centred around 578 nm. They attributed it to the surface defects without an assignment of chemical origin.

Count traces and histograms shown in Figure 6c,d reveal that the defect blinks between two distinct “off” and “on” states from 0 to 8 min and remains relatively stable between 8 and 17 min, followed by a permanent bleaching from 17 to 20 min. The change in the maximum count rate just before and after the 8 min, 4 × 10^4^ c/s and 2.6 × 10^4^ c/s, suggests that the first excited state was permanently photoionised and that after 8 min the defect had changed to a new electronic configuration. The data shown in Figure 6 was the only defect found that exhibited single-photon emission in the anatase nanoparticles.

The reason for the noisy spectrum and normalised *g^(2)^* was the relatively low maximum count rate. At a pump power of 41.9 ± 0.1 μW, the maximum count rate was 35 × 10^3^ c/s for the single-photon emitter. A low pump power was chosen for two reasons: (1) to be in the low-power regime when calculating the lifetimes; and (2) to prevent inadvertent photobleaching. We observed that many potential circular features would readily photobleach at pump powers of hundreds of microwatts or above.

The coupling efficiency can be increased for both morphologies that exhibit single-photon emission. For defects within thin films, a photonic cavity structure can be engineered around the single emitters and the spontaneous emission rate can be increased via the Purcell effect. TiO_2_ has already been shown to be a photonic cavity material [16,19–21,62–64]. For defects within nanoparticles, the emission rate can be increased by encapsulation with material having a refractive index greater than that of air [65].

The issue of native defects in oxides is generally very complex. While the molecular formation and energy structures of defects have been previously discussed in the literature, a majority of these studies is concerned with catalytic and photocatalytic applications [34,66]. Therefore, the majority of existing research is focussed on obtaining defects that result in absorption in the visible spectral range. Then again, works on defects in TiO_2_ have been scarce. While some works have associated visible luminescence with specific defects [67], the obtained emission spectra are very broad, covering the entire visible spectral range.

At this stage, we cannot attribute a particular defect to the three bands observed. Future work would focus on the identification of the origin of the defects via density functional theory. In addition, the low probability of defects needs to be addressed to increase the number of defects formed during the fabrication process. Also, liquid nitrogen measurements may also reveal stronger signals in the emission peaks by suppressing the contributions due to phonon sidebands.

## Conclusion

This study investigated thin films, single crystals and nanopowders of TiO_2_ via confocal microscopy. For the first time, it has been observed that TiO_2_ defects exhibit antibunching behaviour within thin films and anatase nanoparticles. This shows that TiO_2_ defects are a room-temperature single-photon source. The excited-state and non-radiative lifetimes were found to be within the range of several nanoseconds and tens of nanoseconds, respectively. The fluorescence occurred in the red emission band. The photodynamics of the defects ranged from photostable to blinking between two excited states. Future work would require optimisation of the growth conditions to increase the statistical prevalence of the fluorescent defects. This confocal microscopy study of TiO_2_ morphologies allows for the emission from individual defects to be resolved. This is essential for determining the chemical origin of the defects, which is subject of future work. These results pave the way to progress the studies into TiO_2_ as a material that hosts room-temperature single-photon emitters for practical quantum applications.
